# Surgical uprighting and repositioning of unerupted molars: Features and findings of a retrospective sample

**DOI:** 10.4317/jced.58972

**Published:** 2021-12-01

**Authors:** Roberto Pippi, Luca Luigetti, Alessandra Pietrantoni

**Affiliations:** 1 Associate professor, Department of Odontostomatological and Maxillo Facial Surgery, Sapienza University of Rome, Italy; 2 Private practicioner, Department of Odontostomatological and Maxillo Facial Surgery, Sapienza University of Rome, Italy; 3 PhD student, Department of Odontostomatological and Maxillo Facial Surgery, Sapienza University of Rome, Italy

## Abstract

**Background:**

Surgical uprighting and repositioning have been proposed to obtain a correct alignment of unerupted permanent molars. A retrospective clinical study was performed to verify the effectiveness of these techniques.

**Material and Methods:**

In order for a case to be included in the study, adequate clinical documentation was required, including radiographic imaging before and after therapy. The degree of inclination of each treated molar was evaluated on pre-operative panoramic radiographs.

**Results:**

Fifty-two molars were studied. Molar involvement was more frequent in the mandible (45 cases=86.54%) than in the maxilla (7 cases=13.66%). The mean age of patients treated with completely formed molars was 17.28±2.86, while that of patients with incompletely formed molars was 12.89±1.75. The most frequent position was mesio-angular (39=75%) with a mean inclination of 31.61°±12.9° (range 5.57°-61.26°). Disto-angular molars had a mean inclination of -28.84°±6.49° (range -23.79°- -36.16°). Surgical uprighting was performed in 37 cases (71.15%), while surgical repositioning was performed in 15 cases (28.85%). Three cases were lost during the follow-up. The outcome was positive in the remaining 49 cases. Complications occurred in only 4 surgeries (7.69%).

**Conclusions:**

Surgical uprighting and repositioning are reliable therapeutic solutions for unerupted mandibular molars, with a favorable prognosis.

** Key words:**Molar impacted, surgical repositioning, surgical up-righiting, tooth impacted, tooth unerupted.

## Introduction

Among methods suggested to solve eruption anomalies of molar teeth, surgical uprighting and surgical repositioning were proposed to obtain a correct alignment of unerupted permanent molars with minimal surgical invasiveness, without the need of high patient compliance and rapid when compared to surgical-orthodontic methods (1,2).

**Figure 1:**
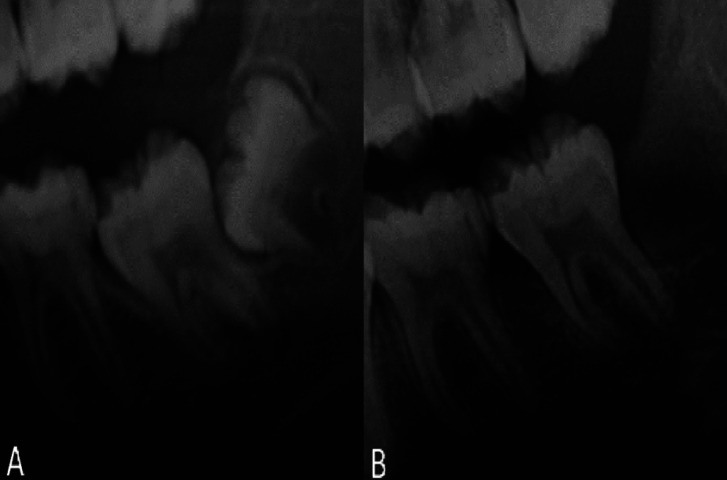
Orthopanoramics of a case treated with surgical uprighting. A) Before the treatment, B) After the treatment.

**Figure 2:**
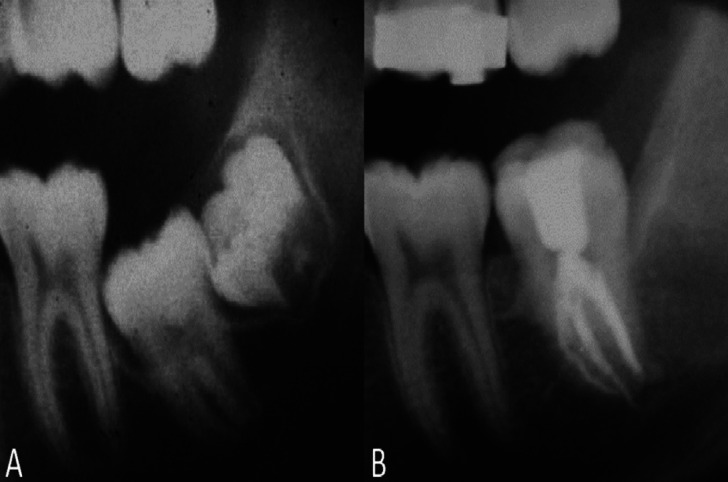
Orthopanoramics of a case treated with surgical repositioning. A) Before the treatment, B) After the treatment, the tooth loss his vitality.

Both these methods consist in modifying the axial inclination of the molar by means of luxation until it is aligned with its physiological eruptive path. They differ from each other in the movement imposed on the molar. In the case of surgical uprighting (Fig. 1A,B) the movement uses the root apex as the fulcrum, so the apex remains in its original position, while in surgical repositioning (Fig. 2A,B) there is a translation of the entire tooth (1-3). This last technique can also be used in cases where the molar is distant from the bone ridge, although it is in a vertical position. Some authors used the term surgical uprighting to define both surgical techniques (4).

A retrospective clinical study was performed to verify the effectiveness of surgical uprighting and surgical repositioning and the incidence of surgical complications associated with these techniques in the treatment of unerupted permanent molars.

## Material and Methods

The study was part of a retrospective study approved by the Ethical Committee with protocol number 3731.

All cases of surgical uprighting and surgical repositioning performed at the Oral Surgery Unit of the Head-Neck Department, Dental Area of the Umberto I Hospital of Rome from 1990 to 2020 were reviewed.

For data collection, an Excel database was created (Microsoft, Redmond, Wash).

In order for a case to be included in the study, adequate clinical documentation was required, including radiographic imaging before and after therapy.

The following data were recorded: age, gender, race, unerupted molars, local etiologic factors, degree of apical formation (complete/incomplete), molar crown coverage and axial inclination, local associated pathologic conditions, treatment modality, related therapies, treatment complications, and outcome.

To perform surgical uprighting and repositioning, it was necessary that the space in the dental arch be adequate for positioning of the molar crown and that no extrusion of opposing molars be present.

**Figure 3:**
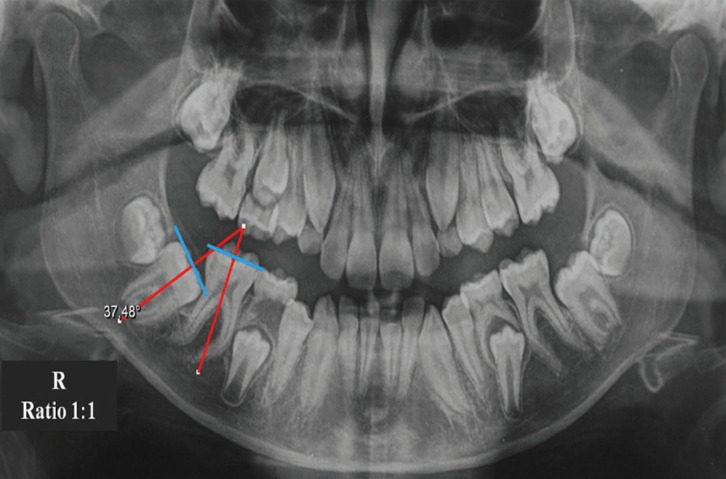
Lower right second molar inclination. The longitudinal axis of first and second molars (red lines) are perpendicular to the respective occlusal planes (blue lines).

The degree of inclination of each treated molar was evaluated on the pre-operative panoramic radiograph by measuring the angle between its long axis and that of the adjacent mesial tooth. To this end, the tangent to the cusps of each of the two teeth and the perpendiculars passing through the centre of the crown and the roots of both teeth were traced, which represented their respective long axis (Fig. 3). The angle, expressed in degrees, between these axes was used as the inclination, conventionally positive for the mesio-inclination, and negative for the disto-inclination.

Tooth movement was always achieved by means of a straight elevator which was perpendicularly inserted into the interdental space between the molar which had to be moved and the mesial tooth in the case of mesio-angular position, and distally, in the case of disto-angular position. The elevator was then gently rotated so that the lower edge of its working end lifted the tooth to be moved.

The outcome of treatment was evaluated as positive when the molar was reported as being correctly aligned in the dental arch at least six months after surgery, without pathological mobility and without any peri-radicular radiolucency, in the case of endodontic treatment due to loss of vitality.

## Results

The sample included 36 patients, 23 of which were females (63.89%) and 13 were males (36.11%). Surgical procedures were always successful. The unerupted molars were 52, since 16 patients had 2 unerupted molars (44.44%), of which 34 (65.38%) in the females and 18 (34.62%) in the males. The mean age of patients at the time of treatment was 14.08±2.86 (range 7-22), slightly lower in the females (13.94±2.73) than in the males. Molar involvement was found to be more frequent in the mandible (45 cases=86.54%) than in the maxilla (7 cases=13.66%). Only 1 first molar was involved (Table 1).

**Table 1:**
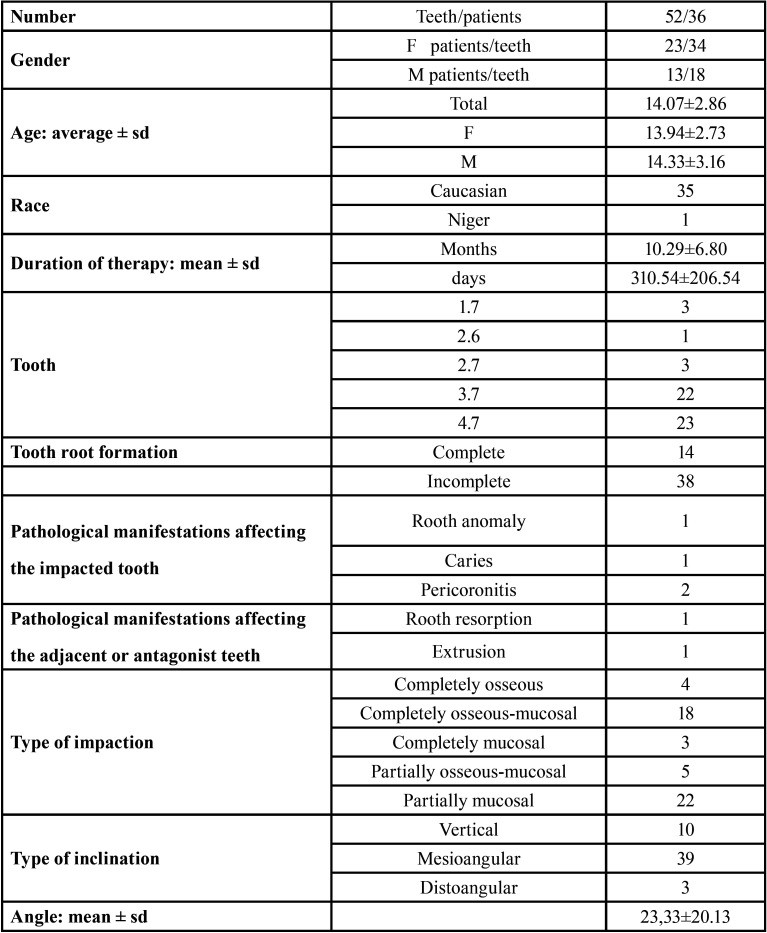
Overall data of the study sample.

The sample was almost completely represented by Caucasians (35/36=97.22%). Out of the 52 treated molars, 38 had an incomplete (73.07%) and 14 (26.92%) a complete apex formation. It was possible to define the outcome of surgeries in 49 cases, since 3 out of the 52 patients were lost during follow-up. The result was considered positive in all 49 patients (100%).

The mean age of patients treated with completely formed molars was 17.28±2.86, while that of patients with incompletely formed molars was 12.89±1.75.

Pathological conditions were present in 6 cases (11.53%), 4 of which involved incompletely formed treated molars (1 root anomaly, 1 cavity, and 2 pericoronitis), 1 involved the contiguous molar (root resorption), and 1 involved the opponent tooth (extrusion).

The most frequent position was mesio-angular (39=75%) with a mean inclination of 31.61°±12.9° (range 5.57°-61.26°). Disto-angular molars had a mean inclination of -28.84°±6.49° (range -23.79°- -36.16°).

Surgical uprighting was performed in 37 cases (71.15%), 2 of which were associated with a conductive alveolectomy. Surgical repositioning was performed in 15 cases (28.85%), only 1 of which was associated with the treatment of a local pathological condition that hindered normal eruption (slipping of the third molar bud).

An additional surgical procedure (germectomy or third molar extraction, cystectomy, marsupialization, or extraction of another tooth) was associated in 44 cases (84.61%), and in 7 cases orthodontic therapy for other reasons (13.46%) was also performed.

Complications were found in only 4 surgeries (5.77%), 1 of which had loss of vitality with subsequent root canal treatment, 1 post-surgical inflammation and, finally, 1 root apex fracture which, however, did not lead to the loss of vitality of the involved molar during the 18-month follow-up.

## Discussion

Impacted second molars are usually diagnosed between 10 and 14 years of age (3,5). This could be explained by the association between eruption disorders of molars and puberty-related bone growth and hormonal changes, which are involved in the dental eruption process. The slight delay between the age of physiological second molar eruption and the diagnosis of impaction is possibly due to the low incidence of impaction-related complications and symptoms, affecting the impacted teeth or the adjacent ones (6), which, even in the present study, were present in only 6 out of the total 52 cases (11.54%).

The mean age of patients in the present sample was 14.08±2.86 (range 7-22), slightly higher than that reported by most authors range (10-17) (1-3,7-9). Pogrel *et al*. (3) suggested that the prognosis of treatment is better if the patient is treated between 12 and 13 years of age, before vertical growth stops. Some authors have also suggested that the best time for surgical repositioning is before root formation is completed, in order to simplify the procedure and improve long-term prognosis (2,3,10). However, Valmaseda-Castellón *et al*. (6) and Davis *et al*. (8) reported successful results after this age (mean age: 17.3, range: 14-20 (6); mean age: 13, range: 10-17 (8), as was found in the present study.

The highest incidence of second molar impaction (51/52), as well as the greater incidence of mandibular impacted molars (86.54%), support the findings reported by other authors (6,11), and may be due to the later development of the maxillary third molar (12).

Many authors (9,12-15) reported a higher incidence of unilateral, compared to bilateral, molar impaction reaching up to 81.25% (9), whereas, according to Cassetta *et al*. (42.5%) (16) and Caminiti *et al*. (4), the present study found a very high incidence of bilateralism (16/36=44.44%), although Shapira *et al*. (15) found a different incidence of unilateral impaction in the Israeli population (73%) and in the Chinese-American population (55%), suggesting that a genetic predisposition can be responsible for this feature.

The present study did not find differences as far as side of impaction is concerned, whereas Varpio and Wellfelt (13) found the right side to be more frequently involved than the left one while Cho *et al*. (17) found the opposite to be true. Shapira *et al*. (15) did not find any differences in the Israeli sample whereas they found a left-side prevalence (67%) in the Chinese-American sample.

According to Cho *et al*. (17), and contrarily to Varpio and Wellfelt (13), the present study found a female prevalence (patients=23/36; unerupted molars=34/52), while other authors did not observe any gender prevalence (15,18-19).

The presence of more than one unerupted molar was found in 44.4% of cases according to Valmaseda-Castellòn (6), and such findings could be genetically determined since Vedtofte *et al*. (20) found that eruption anomalies of mandibular second molars were associated with a particular craniofacial morphology, featured by predominance of skeletal class II, low maxillary incisor inclination, low mandibular angles, and mandibular prognathism.

Shapira *et al*. (15) found that all mesially impacted second molars had significant differences between impaction and non-impaction sides. The distance between the first molar and the ramus, for example, the available space for the second molar, was consistently smaller on the impaction side.

In a previous study by Evans (21), a close association was found between the unilateral impaction of the second mandibular molar and the mandibular midline shift towards the impacted tooth, resulting in arch-length deficiency on that side.

In line with previous studies (12-15), the present study showed that teeth were more frequently mesio-inclined, and this is possibly since during their initial development all mandibular molars are mesially inclined (22). However, this result is in contrast with that reported by Valmaseda-Castellòn *et al*. (6), who found a clear predominance of the vertical position.

The loss of vitality occurred in only 1 case. It was a lower right second molar, partially impacted with a 39° mesio-inclination, treated with surgical uprighting in a 14-year-old female. It is possible that the association between the patient’s more advanced age with the relative greater degree of root development and the rather high degree of inclination, caused a compressive trauma of the periapical vascular-nerve bundle causing tooth pulp necrosis, although these features (age>14 years, inclination>30°, surgical uprighting) were also present in another 7 cases in which no serious complications (fracture and/or loss of vitality) occurred.

Root fracture only occurred during 1 case of surgical repositioning of a left lower second molar in an 18-year-old male, totally and vertically bony impacted, whose anatomy was featured by a hook on the mesial root apex, already evident in the OPG, and which represented a high-risk situation for such a complication.

According to previous studies (2-4,6,23,24), surgical uprighting and repositioning were therefore found to be safe and predictable surgical techniques for the retrieval of impacted molars, with a good prognosis and minimal post-operative complications, such as pulp necrosis and root fracture. Due to the 100% positive results and the low incidence of complications, an inclination degree ranging between -36.16 and 61.26 (24.71°±18.35°) was found to not be related to the risk of failure or severe complications in the present study.

Although a higher incidence of complications was previously found to be associated with surgical repositioning (2,3,6,23,24) due to the translation which it involves, no complications occurred in the present sample during this kind of surgery.

Third molar extraction was performed in 43 out of 51 second molar surgeries (84.31%). Third molar extraction benefit during surgical uprighting or repositioning is not unanimously acknowledged. Although it appears useful in obtaining more space for second molar distal luxation, the latter is not actually always necessary (4,9,12,25). However, several authors (2,3) have suggested prophylactic extraction of third molars in all cases of surgical uprighting or repositioning of second molars since third molars are unlikely to erupt in the correct position. However, it seems more reasonable to perform third molar extractions when the repositioned second molar is sufficiently sTable so that if the second molar cannot be conserved, as in the case of a root fracture, the third molar can be used to replace it (9). Obviously, contextual extraction is necessary if the third molar clearly impedes second molar shifting, free from any further resistance (4,23).

An additional technical aspect which should be addressed is the stabilization of the shifted molar. In the present study, stabilization was performed in 20 out of 52 shifted molars, using different methods such as the insertion in the mesial interdental space of a coiled brass wire, a bone or enamel wedge or the application of a reinforced composite splinting (Table 2). In this regard, some authors (26,27) have argued that the use of additional methods to stabilize the tooth is rare. However, autogenous bone or bone substitutes (4,8,27), tooth splinting with buccally bonded brackets (5,18) or wires (3,9), or surgical packs (1,4) have sometimes been used. In order to guarantee successful treatment, features such as patient adulthood, complete root development, presence of roots with high degrees of divergence or marked curvature, and high degrees of tooth inclination should be considered in choosing surgical uprighting and repositioning, also taking into consideration that these techniques are often indicated, due to their shorter duration, as alternatives to conventional orthodontic treatments, especially in older patients who do not require orthodontic therapy for other purposes.

**Table 2:**
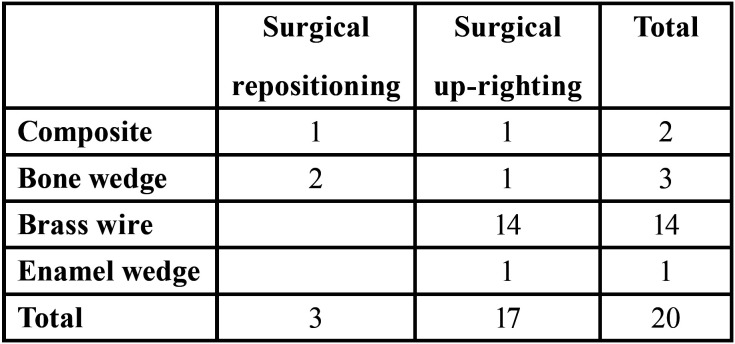
Methods of tooth stabilization.

In conclusion, surgical uprighting and repositioning represent reliable therapeutic solutions for unerupted mandibular molars, with a favorable prognosis, although they are not completely free from complications which are, however, minor and whose incidence can be reduced by an adequate assessment of the anatomic-topographical features of the unerupted tooth.
